# The Impact of Nirsevimab on Bronchiolitis‐Related Hospitalizations: A Multicenter Italian Retrospective Comparative Study

**DOI:** 10.1002/ppul.71500

**Published:** 2026-02-09

**Authors:** Sergio Ghirardo, Barbara Madini, Nicola Ullmann, Alessandro Zago, Michele Ghezzi, Enza D'Auria, Marta Minute, Stefano Martelossi, Anna Chiara Vittucci, Sebastian Cristaldi, Sara Procoli, Francesca Castelletti, Vincenzo Guaia, Alessia Rocchi, Beatrice Andrenacci, Francesco Maria Risso, Andrea Trombetta, Luca Barchi, Simone Foti Randazzese, Sara Manti, Eloisa Gitto, Virginia Mirra, Paolo Siani, S. Salvatore Aversa, Alessandro De Fanti, Maria Francesca Patria, Alessandro Amaddeo, Egidio Barbi, Renato Cutrera

**Affiliations:** ^1^ Department of Medicine, Surgery and Health Sciences University of Trieste Trieste Italy; ^2^ Institute for Maternal and Child Health IRCCS “Burlo Garofolo” Trieste Italy; ^3^ Fondazione IRCCS Cà Granda Ospedale Maggiore Policlinico, S.C. Pediatria Pneumoinfettivologia Milan Italy; ^4^ Respiratory and Cystic Fibrosis Unit, Academic Pediatric Department Bambino Gesù Children Hospital, IRCCS Rome Italy; ^5^ Department of Pediatrics Buzzi Children's Hospital Milan Italy; ^6^ Department of Biomedical and Clinical Sciences University of Milan Milan Italy; ^7^ Unit of Pediatrics Ospedale Regionale Ca Foncello Treviso Treviso Italy; ^8^ Pediatric Unit, Pediatric Emergency Department Bambino Gesù Children's Hospital, IRCCS Rome Italy; ^9^ Neonatal Intensive Care Unit, Children's Hospital ASST Spedali Civili Brescia Italy; ^10^ Pediatric Unit Azienda USL‐IRCCS di Reggio Emilia Reggio Emilia Italy; ^11^ Pediatric Unit, Department of Human Pathology in Adult and Developmental Age “Gaetano Barresi” University of Messina Messina Italy; ^12^ Neonatal and Pediatric Intensive Care Unit, Department of Human Pathology in Adult and Developmental Age “Gaetano Barresi” University of Messina Messina Italy; ^13^ Pediatric Unit, Department of Chronic and Multifactorial Diseases AORN Santobono‐Pausilipon Naples Italy

## Abstract

**Introduction:**

Bronchiolitis is the leading cause of infant hospitalizations worldwide, primarily driven by respiratory syncytial virus (RSV).

**Methods:**

This multicenter retrospective comparative study assessed the impact of immunoprophylaxis with nirsevimab against RSV on bronchiolitis‐related hospitalizations in Italy during the 2024–2025 winter season.

**Results:**

Data from nine Italian hospitals showed a substantial 48% decrease in bronchiolitis admissions compared to the previous season (438 vs. 832 admissions). Among hospitalized infants, only 23% had received immunoprophylaxis. RSV positivity dropped significantly among immunized patients (48%) versus non‐immunized (73%, *p* < 0.0001), with fewer RSV‐related coinfections.

**Discussion:**

While indicators of illness severity—such as ICU admission, respiratory support needs, and complications—were generally lower, no statistically significant differences in disease course were observed between immunized and non‐immunized hospitalized infants. A shift in viral epidemiology was noted, with a reduction in RSV dominance and increased detection of rhinovirus and enterovirus, suggesting a pathogen replacement effect. The 2024–2025 season also saw a lower intubation rate (< 0.5%), pointing to an overall milder disease course.

**Conclusions:**

This study supports the effectiveness of nirsevimab in reducing RSV‐associated hospitalizations and reshaping the virological landscape of bronchiolitis in Italy. Early and widespread implementation of immunoprophylaxis is recommended to maximize public health benefits.

## 1 Introduction

Bronchiolitis has traditionally been the leading cause of hospital admissions in infants under 1 year of age in high‐income countries [1]. Bronchiolitis hospitalizations vary throughout the year according to seasons, usually peaking in November‐December in Italy, in line with the seasonality of respiratory syncytial virus (RSV), which is the main driver of this clinical condition [2]. The COVID‐19 pandemic disrupted this well‐known pattern: social distancing and lockdown measures caused an unprecedented reduction in bronchiolitis incidence and severity during the 2020–2021 season, as documented in our previous multicenter and single‐center studies [3, 4]. The year immediately following the pandemic saw a return to hospitalization levels slightly higher compared to the prepandemic period with an escalation in the level of care [5]. However, in 2022–2023 winter season, there was a record number of admissions—nearly doubled compared to the prepandemic period—along with an increased need for respiratory support, placing a greater burden on pediatric intensive care units and leading to higher intubation rates [6, 7]. Numbers and severity in Italy went back to normal during the last winter season 2023–2024 [8]. Population‐based surveillance studies have similarly documented substantial year‐to‐year variability in RSV‐related hospitalization incidence, highlighting the marked post‐pandemic rebound and subsequent decline observed across Europe [9, 10].

In this context, nirsevimab—a long‐acting fully humanized monoclonal IgG1 antibody targeting the prefusion F protein of RSV—represents a promising advance in passive immunization [11]. When compared to palivizumab, a humanized monoclonal antibody [12], nirsevimab exhibits a longer half‐life allowing a single administration as opposed to monthly during the RSV season for palivizumab [13] Palivizumab is labeled for high‐risk populations [14, 15, 16]. But nirsevimab efficacy should be carefully monitored in the real‐world due to the per se rapidly evolutive bronchiolitis epidemiology after COVID‐19 related lockdown and first impact containment measures [17]. It has been proven effective in reducing bronchiolitis‐related hospitalizations in Spain [9, 18, 19]. In Italy, nirsevimab immunization was firstly introduced in the Valle d'Aosta region for newborns born in the 2023–2024 season. There, its effectiveness was reported to be complete, with no hospitalizations among infants who received nirsevimab, compared to 8.3% among those who did not [20]. Nirsevimab was approved in 2024 in Italy, and its administration began in a staggered manner. The immunoprophylaxis is proposed to all neonates and young infants entering their first respiratory syncytial virus (RSV) season irrespectively from gestational age or underlying risk factors. For infants born before the onset of the RSV season—typically considered “off‐season births” (e.g., April–September)—it is recommended that Nirsevimab be administered in October prior to the beginning of RSV circulation.

In Italy children eligible for Nirsevimab in their second RSV season (up to 24 months) are children with prematurity < 35 weeks, moderate–severe bronchopulmonary dysplasia, hemodynamically significant congenital heart disease, severe immunodeficiency, neuromuscular disorders, cystic fibrosis with pulmonary involvement, chronic lung disease, or complex conditions requiring airway or ventilatory support. This study aims to evaluate the real‐world impact of nirsevimab on bronchiolitis hospitalizations during the 2024–2025 winter in Italy. By comparing bronchiolitis‐related hospitalizations with historical data from previous years, we seek to assess the effectiveness of nirsevimab in reducing the burden of bronchiolitis in infants. We hypothesized that the introduction of nirsevimab immunoprophylaxis would lead to a significant reduction in bronchiolitis‐related hospitalizations, a lower rate of RSV positivity among hospitalized infants, and a milder clinical course.

## 2 Methods

We conducted a multicenter retrospective comparative study to evaluate the bronchiolitis hospitalization trends across the years from the 2019–2020 to the 2024–2025 winter season and a cross‐sectional analysis of data to evaluate the impact of immunoprophylaxis with nirsevimab on bronchiolitis‐related hospitalizations among infants in Italy. The study included data from nine Italian hospitals geographically distributed across the country: Bambino Gesù Children's Hospital IRCCS, Rome; Vittore Buzzi Children's Hospital, Milan; Neonatal Intensive Care Unit, ASST Spedali Civili, Brescia; IRCCS “Burlo Garofolo,” Trieste; Ca' Granda Ospedale Maggiore Policlinico, Milan; Ospedale Regionale Ca Foncello, Treviso; AOU “G. Martino,” Messina; Santa Maria Nuova di Reggio Emilia; AORN Santobono‐Pausilipon, Naples, please refer to Supporting Information S1: Figure 1 for centers localization across the country. Data were retrospectively extracted from clinical records, including both electronic and paper medical records for all infants under 1 year of age hospitalized from September 1st to March 31st hereinafter referred to as the “winter season” for each year starting from 2019. We considered eligible and included all and only consecutive patients with clinical records reporting a discharge diagnosis of bronchiolitis. We collected background data including hospital of admission, age, sex, gestational age, birth weight, and underlying comorbidities at the onset of symptoms. Comorbidities were grouped into five categories based on classifications described in the literature [21]: ex‐preterm, neuromuscular disease, heart disease/defects, chronic lung disease, immunodeficiency. The exposed group included infants who had received immunoprophylaxis with nirsevimab or palivizumab prior hospitalization; the unexposed group included those who had not. We collected data on immunoprophylaxis with nirsevimab and palivizumab: whether prophylaxis was it performed, the date and the number of doses of the latter. Virologic results were recorded, noting whether RSV was detected via polymerase chain reaction (PCR), either through an RSV‐specific assay or a multiplex PCR panel. When multiplex PCR tests were performed on nasal or pharyngeal swabs or aspirates, results for other detected viruses were also documented. To assess the course of the disease, we recorded the dates of hospitalization and discharge, whether admission to the intensive care unit (ICU) occurred and the length of ICU stay, as well as whether low‐flow oxygen, high‐flow nasal cannula (HFNC), continuous positive airway pressure (CPAP), non‐invasive ventilation (NIV), or invasive respiratory support was initiated, along with the number of days each was used. The data were collected between the 1st and 30th of April. No additional follow‐up beyond hospitalization was conducted thus loss to follow‐up was not applicable. To limit misclassification bias, we relied on discharge diagnoses and applied a consistent case definition: patients were included only if they had standardized ICD‐9 discharge codes 466.11 (“Acute bronchiolitis due to Respiratory Syncytial Virus”) or 466.19 (“Acute bronchiolitis due to other infectious organisms”). To further reduce this bias, data were extracted exclusively from verified medical records. Data extraction and tabulation were performed using a uniform protocol across all centers, and ambiguous cases were reviewed by local investigators cross‐checking between clinical notes and laboratory records when available. Variability between centers was addressed by analyzing data both in aggregate and stratified by region. Missing data were < 0.5% for all variables. Given this extremely low proportion and the fact that missingness was largely attributable to incomplete documentation rather than selective absence of information, missing values for comorbidities and prophylaxis status were treated as absence, following standard practice in retrospective hospital‐based datasets. No formal sensitivity analyses (e.g., complete case analysis or multiple imputation) were performed because the negligible amount of missing data made such procedures unlikely to influence the results. The exact number of missing observations for each variable is reported in the corresponding tables of the supporting material. No imputation was performed for missing data. This deterministic approach is unlikely to introduce meaningful bias given the very low proportion of missing data and the retrospective nature of the dataset.

### 2.1 Statistical Analysis

Discrete variables were reported as counts and percentages of the total. We assessed the distribution of continuous variables both graphically and using the Shapiro–Wilk test. Variables with a normal distribution were reported as mean and standard deviation, while non‐normally distributed variables were presented as median with first and third quartiles. We compared the winter seasons and region to region using one‐way ANOVA. For non‐parametric group comparisons, we used the Kruskal–Wallis ANOVA, followed by post hoc multiple comparisons with Bonferroni correction at an alpha level of 0.05. We compared the 2024–2025 winter season with the preceding season using the Chi‐square test for categorical variables and the Student's *t*‐test for continuous variables. Length‐related outcomes, such as duration of hospital stay and days requiring various forms of respiratory support, were analyzed using the log‐rank test. A *p*‐value of < 0.05 was considered statistically significant. During the 2024–2025 winter season, we compared disease severity parameters (length of stay, ICU need, ICU length of stay, respiratory support) between patients who received immunoprophylaxis and those who did not, using the Chi‐square test or log‐rank test where appropriate. A formal sample size calculation was not performed, as the study included the total population of eligible infants hospitalized across nine centers over six seasons, ensuring broad representativeness. Prematurity, birth weight, and age at admission were included as covariates in the descriptive and comparative analyses to account for potential confounding.

The primary outcome was the number of hospitalizations for bronchiolitis from September 1 to March 31 of the 2024–2025 season, compared to the previous 4 years. Secondary outcomes included severity measures between patients who received immunoprophylaxis and those who did not, in terms of hospital and ICU length of stay, as well as the need for respiratory support.

## 3 Results

During the 2024–2025 winter season, a total of 438 infants were admitted due to bronchiolitis across the nine hospitals involved in the study. In comparison, there were 832 admissions during the same period in 2023–2024, 1161 in 2022–2023, 700 in 2021–2022, 43 in 2020–2021, and 512 in 2019–2020. During the 2024–2025 and 2020‐2021 winter seasons, patients were statistically significantly older compared to previous winter seasons (*p* < 0.0001). In 2020–2021 and 2021–2022 winter seasons hospitalizations occurred statistically earlier compared with the other winter seasons considered. See Table 1 for background data.

**TABLE 1:** Patient background data at the time of hospitalization.

	2024–2025	2023–2024	2022–2023	2021–2022	2020–2021	2019–2020	
Number of patients	438	832	1161	700	43	512	
Gestational age (weeks)	38 (2.5)	38 (2.5)	38 (2.4)	39 (2.2)	38 (2.4)	38 (2.4)	*p* = 0.37
Birth weight (grams)	3,148 (618)	3,188 (601)	3,201 (609)	3,263 (553)	3,319 (614)	3,234 (602)	*p* = 0.04
Age at admission (days)	135 (104)	96 (79)	87 (76)	75 (70)	129 (107)	91 (81)	*p* < 0.0001
Days from symptoms onset and admission	3 (2–4)	3 (2–4)	3 (2–4)	3 (2–4)	2 (1–5)	2 (1–4)	*p* = 0.09
Days from 1st September to admission	118 (96–146)	131 (109–161)	109 (87–129)	82 (68–100)	75 (42–152)	125 (106–159)	*p* < 0.0001
Patients who received immunoprophylaxis	100 (23%)	18 (2%)	23 (2%)	13 (2%)	2 (5%)	11 (2%)	*p* < 0.0001
Patients with comorbidities	72 (16%)	135 (16%)	242 (21%)	121 (17%)	7 (16%)	116 (23%)	*p* = 0.012

*Note:* Data are reported as mean and standard deviation (in parentheses) if normally distributed, or as median with the first and third quartiles (Q1–Q3) if not normally distributed.

The birth weight was slightly statistically significantly lower during 2021–2022 winter season and the age at admission was higher during 2020–2021 and 2024–2025 winter seasons. prematurity did not differ significantly across groups no multivariable models were applied.

Prevalence of immunoprophylaxis varied significantly by region (*p* = 0.001), ranging from 52 out of 146 hospitalized patients (36%) at Ospedale Maggiore Policlinico and Buzzi Children's Hospital to 22 out of 199 (11%) at Bambino Gesù Children Hospital IRCCS. Potential confounders such as prematurity, birth weight, and age at admission were assessed and found not to differ significantly across the winter seasons analyzed, suggesting limited confounding in comparisons of disease burden and severity over time. Missing data were less than 0.5% for each parameter considered. Missing data were considered as absence of the respective condition or exposure, and percentages were calculated based on available data. Since missing data were excluded from the study, sensitivity analyses (complete case analysis, multiple imputation, and extreme value imputation) were conducted to assess the impact of missing data on the incidence of RSV‐related bronchiolitis, hospitalizations, and ICU admissions during the first winter season following the introduction of nirsevimab in Italy, compared to previous years. As this was a retrospective study with no follow‐up beyond hospitalization, loss to follow‐up was not applicable. Given the extremely low rate of missing data (< 0.5%) and consistent data collection protocols across centers, we did not perform formal sensitivity analyses, as the potential impact on outcomes was considered negligible.

### 3.1 Virological Results

A total of 429 (98%) subjects were tested for viruses during the 2024–2025 winter season. This compares to 807 (97%) subjects in 2023–2024, 1086 (93%) in 2022–2023, 655 (94%) in 2021–2022, 38 (88%) in 2020–2021, and 479 (93%) in 2019–2020. In terms of RSV positivity detected by RSV‐specific assays or PCR panels, 296 (69%) tested positive in 2024–2025, 680 (84%) in 2023–2024, 919 (85%) in 2022–2023, 581 (89%) in 2021–2022, 6 (16%) in 2020–2021, and 381 (79%) in 2019–2020.

A total of 381 subjects (87%) were tested with a PCR panel during the 2024–2025 winter season, compared to 759 (91%) in 2023–2024, 922 (79%) in 2022–2023, 545 (78%) in 2021–2022, 37 (86%) in 2020–2021, and 465 (90%) in 2019–2020.

Overall, rhinovirus was the most frequently detected virus, alongside RSV. However, rhinovirus was statistically significantly more common during the 2020–2021 and 2024–2025 winter seasons (*p* < 0.001). Enterovirus showed a similar pattern but was only more common during the 2024–2025 winter season (*p* < 0.001). Coronavirus was statistically significantly less frequent during the 2019–2020 and 2020–2021 winter seasons (*p* = 0.0004). During the 2023–2024 winter season, there was a slight but statistically significant increase in the detection of adenovirus. Finally, during the 2020–2021 winter season, a statistically significantly higher percentage of patients tested negative for all viruses checked (*p* < 0.001). Refer to Table 2 for the viral of PCR panels considering that every panel used included RSV.

**TABLE 2:** Virological results of PCR panels.

	2024–2025	2023–2024	2022–2023	2021–2022	2020–2021	2019–2020	*p*
PCR panel tested subjects (%)	381 (87%)	759 (91%)	922 (79%)	545 (78%)	37 (86%)	465 (90%)	
Rhino‐virus (%)	149 (39%)	163 (21%)	224 (24%)	123 (23%)	23 (62%)	83 (18%)	< 0.001
Entero‐virus (%)	50 (13%)	43 (6%)	75 (8%)	11 (2%)	2 (5%)	18 (5%)	< 0.001
Corona‐virus (%)	40 (10%)	53 (7%)	66 (7%)	32 (6%)	1 (3%)	13 (3%)	0.0004
Meta‐pneumo‐virus (%)	29 (8%)	53 (7%)	57 (6%)	34 (6%)	0	29 (6%)	0.56
Boca‐virus (%)	22 (6%)	36 (5%)	29 (3%)	19 (3%)	0	17 (3%)	0.15
Adeno‐virus (%)	13 (3%)	39 (5%)	25 (3%)	13 (2%)	0	16 (3%)	0.046
Para‐influenza virus (%)	15 (4%)	22 (3%)	39 (4%)	30 (5%)	3 (8%)	20 (4%)	0.22
Influenza virus (%)	14 (4%)	33 (4%)	45 (5%)	12 (2%)	0	17 (4%)	0.13
Other (%)	5 (1%)	11 (1%)	16 (2%)	6 (1%)	0	5 (1%)	0.85
No virus detected (%)	2 (0%)	19 (2%)	46 (5%)	20 (4%)	8 (21%)	39 (8%)	< 0.001

*Note:* Number of patients testing positive for each virus. Percentages are calculated based on the total number of patients tested using a polymerase chain reaction (PCR) panel. RSV stays for respiratory syncytial virus. *P* values were calculated using the Chi‐square test.

The coinfection rate was significantly higher after the pandemic (*p* < 0.0001). Interestingly, the number of patients with a coinfection involving RSV was significantly lower during the 2024–2025 winter season (*p* < 0.0001). Specifically, the number of patients with a coinfection involving two viruses, including RSV, was significantly lower during the 2024–2025 winter season. However, the number of patients with a three‐ or four‐virus coinfection was higher overall. Notably, only four patients who received immunoprophylaxis developed a three‐virus coinfection involving RSV, and none developed a coinfection involving four or more viruses with RSV.

Refer to Table 3 for further data about coinfection

**TABLE 3:** Coinfections rates.

	2024–2025	2023–2024	2022–2023	2021–2022	2020–2021	2019–2020	*p*
Coinfections (%)	148 (39%)	261 (34%)	325 (35%)	172 (31%)	5 (11%)	113 (24%)	< 0.0001
Coinfections with RSV (%)	95 (64%)	234 (90%)	267 (82%)	147 (85%)	2 (40%)	97 (86%)	< 0.0001
Coinfections without RSV (%)	53 (36%)	27 (10%)	58 (18%)	25 (15%)	3 (60%)	16 (14%)	< 0.0001
2 viruses (with RSV)	93 (57)	206 (185)	249 (199)	145 (125)	3 (1)	96 (82)	< 0.01/0.0001
3 viruses (with RSV)	41 (32)	47 (43)	68 (62)	26 (19)	2 (1)	13 (13)	0.001/0.001
4 or more viruses (with RSV)	10 (6)	5 (5)	7 (5)	3 (3)	0	2 (2)	0.006/0.3

*Note:* Coinfection rates are reported as percentage of the number of patients tested with a PCR panel. The percentage of coinfections with Respiratory Syncytial Virus (RSV) and without RSV is calculated as a percentage of the total number of coinfected patients.

Among 100 patients hospitalized for bronchiolitis who received immunoprophylaxis, 48 (48%) tested positive for RSV by PCR. In contrast, among the 338 patients who did not receive immunoprophylaxis, 248 (73%) were RSV‐positive. This difference is statistically significant (*p* < 0.0001). Furthermore, the coinfection rate among RSV‐positive patients who had received immunoprophylaxis was 13% (13/100), which was significantly lower than the 24% (82/338) observed in non‐immunized patients (*p* < 0.0001).

### 3.2 Severity

Over these six winter seasons, a total of 24,429 hospitalization days and 4100 ICU days were required for the care of patients with bronchiolitis. The overall length of hospital stay was significantly shorter during the 2024–2025 and 2020–2021 winter seasons (*p* = 0.00002). The need for intensive care was also significantly lower in the 2024–2025 winter season (*p* = 0.0019), although the length of ICU stay did not differ significantly. Additionally, the need for respiratory support was significantly lower during the 2019–2020 and 2020–2021 winter seasons compared to the other seasons, with the duration of respiratory support being significantly shorter in the 2020–2021 season (*p* < 0.00001).

Lastly, the number of complications occurring during hospitalization was significantly higher during the 2022–2023 winter season and lower during the 2019–2020 and 2020–2021 winter seasons (*p* < 0.0001). Refer to Table 4 for information on the variation of severity data across the years.

**TABLE 4:** Severity measures.

	2024–2025	2023–2024	2022–2023	2021–2022	2020–2021	2019–2020	*p*
Length of stay (days): Cumulative Per‐person median (Q1–Q3)	2444 5 (3–7)	5,632 6 (4–8)	8179 6 (4–9)	4434 6 (4–8)	200 4 (3–5)	3540 6 (4–9)	< 0.0001
ICU^a^ admission number (percentage)	50 (11%)	142 (17%)	224 (19%)	132 (19%)	3 (7%)	98 (19%)	0.0019
ICU length of stay (days): Cumulative Per‐person median (Q1–Q3)	262 5 (4–7)	833 5 (4–7)	1586 6 (4–8)	806 6 (3–8)	6 1, 2, 3	607 5 (3–9)	0.0889
Respiratory support need number (percentages)	349 (80%)	669 (80%	942 (81%)	579 (83%)	21 (49%)	343 (67%)	< 0.0001
Days on respiratory support: Cumulative Per‐person median (Q1–Q3)	1597 4 (3–6)	3012 4 (3–6)	3769 4 (2–5)	2674 4 (3–6)	81 3 (2–4)	1634 4 (3–6)	< 0.0001
Complications number (percentages)	173 (39%)	276 (33%)	684 (59%)	195 (28%)	7 (16%)	90 (17%)	< 0.0001

*Note:* Numbers report the cumulative number of days, the median length in days and the first and third quartile.

^a^ ICU: Intensive Care Unit. The percentage of patients admitted to ICU, needing respiratory support and complications are calculated out of the total number of patients.

Considering the type of respiratory support, low‐flow oxygen supplementation was used significantly more frequently during the 2021–2022 winter season, although the duration of oxygen use did not change significantly across the winter seasons. HFNC use was significantly lower during the 2020–2021 winter season, and its duration of use was shorter in both the 2024–2025 and 2022–2023 winter seasons. HFNC was also used significantly less frequently during the 2024–2025 winter season (*p* < 0.0001). CPAP/NIV use was statistically significantly lower during the 2020‐2021 winter season (*p* = 0.01). Intubation occurred significantly less frequently during the 2024–2025 and 2020–2021 winter seasons, whereas during the 2022–2023 winter season, the number of intubations was statistically significantly higher (*p* = 0.0007). The duration of mechanical ventilation (MV) did not differ significantly across the winter seasons. Refer to Table 5 for details on the types of respiratory support and their duration of use.

**TABLE 5:** Respiratory support use (HFNC, CPAP/NIV, MV) and days on support.

	2024–2025	2023–2024	2022–2023	2021–2022	2020–2021	2019–2020	*p* value Chi square *p* value ANOVA
Low flow oxygen Number (percentage)	186 (42%)	300 (36%)	441 (38%)	361 (51%)	10 (23%)	215 (42%)	< 0.0001
Low flow oxygen Days median (Q1–Q3)	3 (2–4)	2 (1–4)	2 (1–4)	2 (1–4)	3.5 (2–6)	3 (2–4)	0.11
HFNC*Number (percentage)	252 (57%)	503 (60%)	708 (61%)	385 (55%)	12 (28%)	205 (40%)	< 0.0001
HFNC* Days median (Q1–Q3)	4 (2–5)	5 (3–6)	4 (2–6)	4 (3–6)	3 (2–4)	4 (3–7)	< 0.0001
Room air HFNC (percentage of HFNC)	16 (6%)	67 (13%)	82 (11%)	52 (13%)	2 (17%)	18 (9%)	< 0.0001
CPAP/NIV** (percentage)	75 (17%)	130 (16%)	209 (18%)	94 (13%)	2 (5%)	68 (13%)	0.01
CPAP/NIV** Days median (Q1–Q3)	3 (2–5)	4 (3–5)	3 (2–5)	3 (2–5)	1, 3 days	3 (2–5)	0.19
MV*** (percentage)	2 (0.4%)	21 (2.5%)	41 (3.5%)	7 (1%)	0	13 (2.5%)	0.0007
MV*** Days median (Q1–Q3)	7, 16 days	4.5 (2–7)	6 (3–9)	5.5 (2.5–10)	0	4 (2–6)	0.07
Highest FiO2 median (Q1–Q3)	30% (21–30)	28% (21–35)	30% (21–37)	30% (21–38)	21% (21–30)	25% (21–30)	< 0.0001

*Note:* HFNC: high‐flow nasal cannula; CPAP*: continuous positive airway pressure; NIV*: non‐invasive ventilation; MV**: mechanical ventilation. The table shows the number of patients requiring each type of respiratory support. Percentages are calculated based on the total number of hospitalized patients. The median refers to the number of days on each type of support, followed by the first and third quartiles. The percentage of patients on HFNC in room air refers to those receiving HFNC without oxygen supplementation, calculated as a proportion of all patients receiving HFNC.

### 3.3 Severity Comparison Between Immunized and Non‐Immunized Patients

Subgroup analyses were conducted by comparing patients who received immunoprophylaxis who did not. Overall, 100 out of 438 patients (23%) had received immunoprophylaxis with nirsevimab or had adequate coverage with palivizumab (one case) at the time of admission. In terms of hospital length of stay, immunized patients had a median of 5 days (Q1: 3–Q3: 7), which was the same as non‐immunized patients (median 5 days, Q1: 3–Q3: 7), with no statistically significant difference (*p* = 0.62). Sixteen immunized patients were admitted to the ICU, compared to 34 non‐immunized patients (*p* = 0.10). The ICU length of stay had a median of 5.5 days (Q1: 4–Q3: 8) for immunized patients and 4 days (Q1: 3–Q3: 6) for non‐immunized patients (*p* = 0.14). Regarding respiratory support, one patient in each group required mechanical ventilation (MV). Among the immunized group (*n* = 100), 19% required CPAP/NIV, 52% required high‐flow nasal cannula (HFNC), and 77% received oxygen in any form. In the non‐immunized group (*n* = 338), 16% required CPAP/NIV, 59% HFNC, and 80% received oxygen in any form. None of these differences were statistically significant: CPAP/NIV (*p* = 0.61), HFNC (*p* = 0.66), and oxygen therapy (*p* = 0.68).

## 4 Discussion

Our multicenter study demonstrated that, despite significant regional variability, there was a substantial nationwide reduction in the incidence of RSV‐related bronchiolitis, hospitalizations, and ICU admissions during the first winter season following the introduction of nirsevimab in Italy, compared to the previous year.

The number of hospitalizations decreased by 48% during the 2024–2025 winter season compared to the winter season of the previous year (2023–2024). Although there was already a trend toward reduction in the previous year, it is likely that nirsevimab played a major role in this decline. While the nationwide percentage of theoretically eligible patients who received nirsevimab in Italy remains unknown, it was reported to be 65% in Valle d'Aosta. Data from other regions are still evolving, but according to regional registries, coverage is 77% in Friuli Venezia Giulia and 28% in Reggio Emilia this year. This hypothesis is further supported by the higher age at admission observed this season, as immunization occurred less frequently in older infants due to logistical reasons. In facts, the immunization is more frequently administered at birth, therefore being more effective in providing immunity in first months of life. As a matter of facts; only 11 of the 74 immunized patients were born before the end of August 2024. Thus, nirsevimab protection was more prevalent in younger patients, likely preventing their hospitalization in favor of older, non‐immunized infants.

Virological results varied significantly, particularly during two periods: the strict social distancing measures implemented during the 2020–2021 winter season and the most recent winter season. During the 2020–2021 winter season, rhinovirus, despite its high transmissibility, experienced a slight decrease but notably became the dominant respiratory virus. This was likely due to the widespread use of surgical masks, which effectively limited the spread of other viruses but did little to preempt rhinovirus [22]. In that season, rhinovirus was involved in more than 60% of cases, similar to what has already been reported in the literature [23], whereas in the baseline and subsequent winter seasons, it was found in approximately one out of five cases on average.

Following the introduction of nirsevimab, the prevalence of rhinovirus increased to nearly 40%, and enterovirus more than doubled, with both changes being statistically significant. While the ability of nirsevimab to prevent the spread of RSV is debated [24], it has proven effective in reducing RSV‐related bronchiolitis in our patient population. Consequently, among infants hospitalized for bronchiolitis, other viruses—primarily rhinovirus and enterovirus—are increasingly causing hospitalizations, effectively replacing RSV as the dominant pathogen in this context.

Notably, the only other virus that showed a statistically significant variation was adenovirus, which nearly doubled compared to its baseline percentage during the 2023–2024 winter season. This is a remarkable finding, especially considering reports in the literature of severe adenovirus infections and related complications during that same year [25, 26, 27].

One final consideration regarding our virological results is the relatively high number of patients with negative PCR tests during the 2020–2021 winter season. Specifically, out of 43 patients, we were unable to identify any virus in eight cases. The most likely explanations for this observation are twofold. First, these patients might have been infected with other viruses not covered by our testing panel. These less common viruses are typically present but account for a smaller percentage of cases during a normal winter. Second, it is possible that these eight patients were instances of misdiagnosis, meaning they were incorrectly identified as having infectious bronchiolitis [28, 29]. While the actual number of misdiagnoses was likely consistent across different winter seasons, the percentage of misdiagnosed cases became notably higher during the 2020–2021 winter. This increase in the proportion of misdiagnoses likely occurred because highly effective viral containment measures, such as social distancing and universal mask‐wearing, significantly reduced the number of genuine bronchiolitis cases, making the misdiagnoses a larger fraction of the total.

Regarding coinfections, they occurred less frequently during the 2020–2021 winter season, as expected, due to the lower number of circulating viruses that winter. Outside of that period, the percentage of coinfections remained consistent across the winter seasons. However, the percentage of coinfections involving RSV among infants hospitalized for bronchiolitis decreased significantly after the introduction of nirsevimab, from over 80% to 64%. At the same time, coinfections not involving RSV rose, more than compensating for this reduction. This finding further supports the replacement mechanism, as already established for other pathogens such as influenza [30] or bacterial serotypes [31].

Beyond the already well‐documented reduced severity of bronchiolitis during the 2020–2021 winter season, our data align with previous reports indicating a more severe course in the 2022–2023 winter season [6, 7], characterized by increased length of stay, ICU admissions, complications, and intubation rates. In contrast, the 2024–2025 winter season showed lower utilization of HFNC and a shorter duration of its use, coupled with an increase in the application of low‐flow oxygen. This latter observation may reflect a genuine reduction in disease severity [32], the implementation of the 2022 Italian guidelines on bronchiolitis management [33] or a combination of both factors. We believe that the halving of HFNC use with room air alone further supports the role of guidelines implementation. Perhaps one of the most remarkable findings in support of the reduction of severity is the abrupt reduction in the intubation rate to less than 0.5% during this winter season, immediately following the introduction of nirsevimab.

In our real‐world study, we did not identify any statistically significantly difference in terms of disease course severity (length of stay, ICU admission and length of stay, respiratory support need) between immunized and non‐immunized patients hospitalized for bronchiolitis. These findings might be partially explained by the emerging incidence of non‐RSV viruses causing bronchiolitis, the relatively small simple size of the study, as well as a result of a changed physician's attitude that may have influenced respiratory support need during hospitalization [5]. Second, immunization was more frequent in younger infants and often administered at birth; aside from receiving prophylaxis, these infants would inherently be more prone to severe bronchiolitis. Differential age, timing from dose to illness, and potential waning could therefore reduce observable contrasts between groups among those who were hospitalized despite immunization. Third, power and outcome choice: the number of immunized inpatients was modest, and severity endpoints (ICU admission, ventilation, length of stay) were relatively infrequent, limiting the statistical power to detect small differences. The main limitation of this study is its retrospective nature. Therefore, we could not rule out potential misclassifications of patients in clinical records, the absence of standardized clinical scores, or the influence of patients' decision‐making. Indeed, this is the first study investigating the impact of bronchiolitis‐related hospitalizations in Italy, with data sourced from several densely populated regions (Veneto, Lombardia, Emilia Romagna, Lazio, Campania, Sicilia, and Friuli Venezia Giulia), reflecting a broad and heterogeneous epidemiology across different social contexts and landscapes.

Lastly, because all hospitals included in the study are tertiary‐level institutions and the analysis exclusively involved hospitalized patients, the generalizability of the findings to other settings may be limited. The results of our study support the recommendation to start immunoprophylaxis as soon as possible during the winter season.

## 5 Conclusions

During the 2024–2025 winter season in Italy, we observed a notable reduction in hospitalizations due to bronchiolitis in infants. The disease course appeared milder on average, with a significant decrease in the number of very severe cases. Furthermore, the virological landscape shifted considerably, with RSV—either alone or in co‐infections—playing a much less prominent role. Given the absence of other evident explanations and the concurrent increase in other viruses, such as rhinovirus and enterovirus, these effects strongly suggest the impact of nirsevimab.

## Author Contributions

**Sergio Ghirardo:** investigation, writing – original draft, methodology, validation, writing – review and editing, conceptualization, project administration, formal analysis, data curation, supervision. **Barbara Madini:** investigation, funding acquisition, writing – original draft. **Nicola Ullmann:** supervision. **Alessandro Zago:** investigation, writing – original draft. **Michele Ghezzi:** writing – review and editing, supervision. **Enza D'Auria:** writing – review and editing, supervision, investigation, conceptualization. **Marta Minute:** writing – review and editing, supervision, investigation. **Stefano Martelossi:** investigation, writing – review and editing, supervision. **Anna Chiara Vittucci:** investigation, writing – review and editing, supervision. **Sebastian Cristaldi:** investigation, writing – review and editing, supervision. **Sara Procoli:** investigation, writing – review and editing, supervision. **Francesca Castelletti:** investigation, writing – review and editing, supervision. **Vincenzo Guaia:** writing – review and editing, investigation, supervision. **Alessia Rocchi:** investigation, visualization, writing – review and editing. **Beatrice Andrenacci:** investigation, visualization, writing – review and editing. **Francesco Maria Risso:** investigation, writing – review and editing. **Andrea Trombetta:** investigation, validation, visualization, project administration. **Luca Barchi:** investigation. **Simone Foti Randazzese:** investigation, project administration. **Sara Manti:** investigation, writing – review and editing, supervision, formal analysis, project administration, data curation, visualization. **Eloisa Gitto:** writing – review and editing, investigation, project administration. **Virginia Mirra:** investigation, writing – review and editing. **Paolo Siani:** investigation. **S. Salvatore Aversa:** writing – review and editing, investigation. **Alessandro De Fanti:** investigation, writing – review and editing, project administration. **Maria Francesca Patria:** supervision, project administration. **Alessandro Amaddeo:** visualization, project administration, supervision, data curation, writing – review and editing, conceptualization. **Egidio Barbi:** writing – review and editing, supervision, resources. **Renato Cutrera:** resources, supervision, project administration, visualization, conceptualization.

## Ethics Statement

This study was conducted using de‐identified retrospective data and was granted an exemption from obtaining informed consent by the Institutional Review Board (IRB) of the Institute for Maternal and Child Health – IRCCS “Burlo Garofolo,” in accordance with institutional and national guidelines. All patients had previously provided general consent for the use of their data in retrospective studies and analyses. Ethical Committee approval was not required under Italian law, as per the General Authorization to Process Personal Data for Scientific Research Purposes (Authorization No. 9/2014). According to this regulation, retrospective studies utilizing coded data—where subjects cannot be directly identified—are exempt from requiring ethics approval. [The Italian Data Protection Authority. Available at: https://www.garanteprivacy.it/web/guest/home/docweb/-/docweb-display/docweb/3786078 (Accessed June 2025).

### 1 Conflicts of Interest

The authors declare no conflicts of interest.

## Supporting information


**Figure 1:** geographical localization of the hospitals involved in the study.

**Table 1:** patient background data at the time of hospitalization: number of patients with missing data for each aspect considered. **Table 2:** virological results of PCR panels: number of patients with missing data for each aspect considered. **Table 3:** coinfections rates: number of patients with missing data for each aspect considered. **Table 4:** severity measures: number of patients with missing data for each aspect considered. **Table 5:** Respiratory support use (HFNC, CPAP/NIV, MV) and days on support: number of patients with missing data for each aspect considered.

## Data Availability

The data that support the findings of this study are available on request from the corresponding author. The data are not publicly available due to privacy or ethical restrictions.
