# Supplementary figures and images for: The Impact of Nirsevimab on Bronchiolitis‐Related Hospitalizations: A Multicenter Italian Retrospective Comparative Study
Source: Pediatr Pulmonol. 2026 Feb 9;61(2):e71500. doi: 10.1002/ppul.71500 (PMC12884209; doi:10.1002/ppul.71500)

Figure 1 geographical localization of the hospitals involved in the study


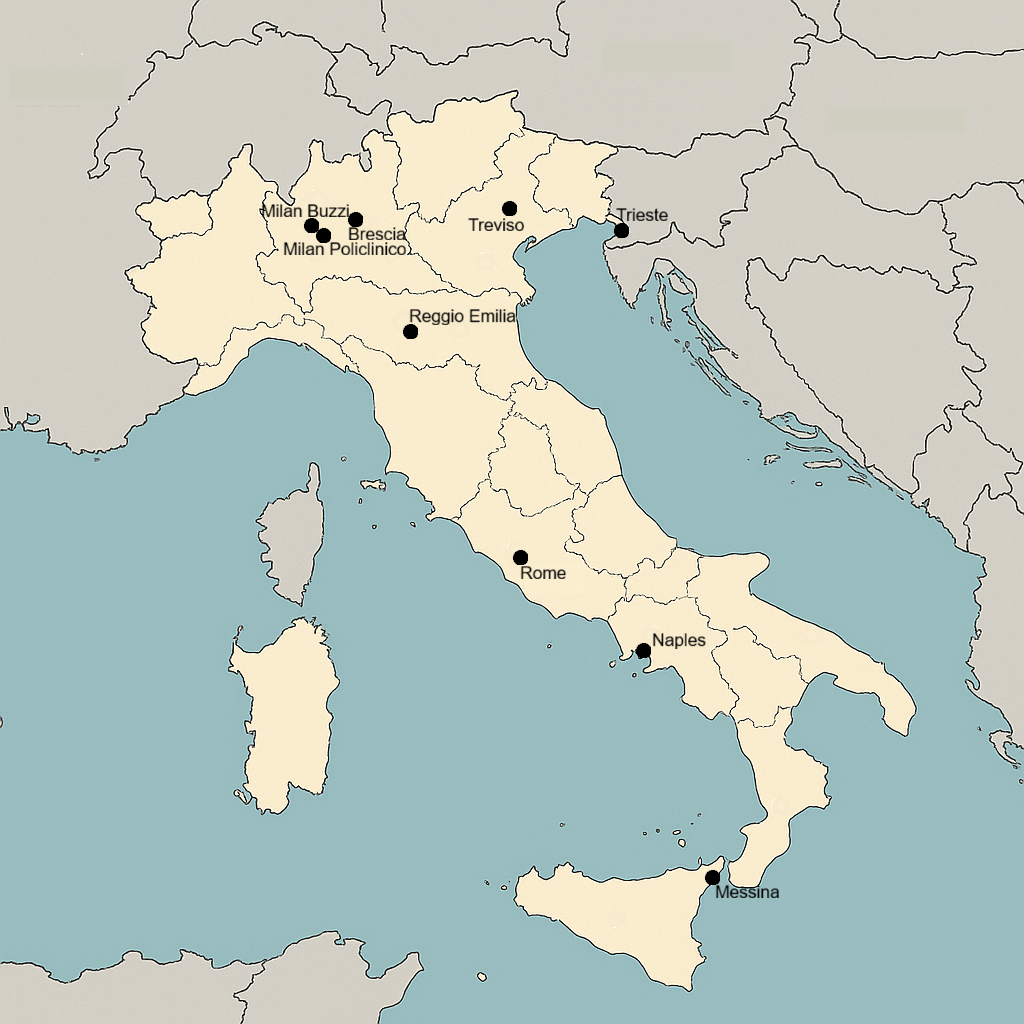

Supplement: Supplementary file 1 — Figure 1: geographical localization of the hospitals involved in the study. [file PPUL-61-0-s001.docx]
