# Supplementary material for: The Impact of Nirsevimab on Bronchiolitis‐Related Hospitalizations: A Multicenter Italian Retrospective Comparative Study
Source: Pediatr Pulmonol. 2026 Feb 9;61(2):e71500. doi: 10.1002/ppul.71500 (PMC12884209; doi:10.1002/ppul.71500)
Supplement: Supplementary file 2 — Table 1: patient background data at the time of hospitalization: number of patients with missing data for each aspect considered. Table 2: virological results of PCR panels: number of patients with missing data for each aspect considered. Table 3: coinfections rates: number of patients with missing data for each aspect considered. Table 4: severity measures: number of patients with missing data for each aspect considered. Table 5: Respiratory support use (HFNC, CPAP/NIV, MV) and days on support: number of patients with missing data for each aspect considered. [file PPUL-61-0-s002.docx]

Table 1 patient background data at the time of hospitalization: number of patients with missing data for each aspect considered

|  | 2024-2025 | 2023-2024 | 2022-2023 | 2021-2022 | 2020-2021 | 2019-2020 |
| --- | --- | --- | --- | --- | --- | --- |
| Gestational age (weeks) | 2 | 3 | 4 | 3 | 0 | 2 |
| Birth weight (grams) | 2 | 4 | 5 | 3 | 0 | 2 |
| Age at admission (days) | 0 | 0 | 1 | 0 | 0 | 0 |
| Days from symptoms onset and admission | 2 | 4 | 4 | 2 | 0 | 2 |
| Days from 1^st^ September to admission | 0 | 0 | 0 | 0 | 0 | 0 |
| Patients who received immunoprophylaxis | 0 | 3 | 3 | 3 | 0 | 2 |
| Patients with comorbidities | 0 | 0 | 0 | 2 | 0 | 2 |

Table 2 virological results of PCR panels: number of patients with missing data for each aspect considered

Table 2 virological results of PCR panels

|  | 2024-2025 | 2023-2024 | 2022-2023 | 2021-2022 | 2020-2021 | 2019-2020 |
| --- | --- | --- | --- | --- | --- | --- |
| RSV | 0 | 0 | 0 | 0 | 0 | 0 |
| Rhino-virus  (%) | 0 | 0 | 0 | 0 | 0 | 0 |
| Entero-virus (%) | 0 | 0 | 0 | 0 | 0 | 0 |
| Corona-  virus (%) | 0 | 0 | 0 | 0 | 0 | 0 |
| Meta-pneumo-virus (%) | 0 | 0 | 0 | 0 | 0 | 0 |
| Boca-  virus  (%) | 0 | 0 | 0 | 0 | 0 | 0 |
| Adeno-  virus  (%) | 0 | 0 | 0 | 0 | 0 | 0 |
| Para-influenza virus (%) | 0 | 0 | 0 | 0 | 0 | 0 |
| Influenza virus (%) | 0 | 0 | 0 | 0 | 0 | 0 |
| Other  (%) | 0 | 0 | 0 | 0 | 0 | 0 |
| No virus detected  (%) | 0 | 0 | 0 | 0 | 0 | 0 |

Table 3 coinfections rates: number of patients with missing data for each aspect considered

|  | 2024-2025 | 2023-2024 | 2022-2023 | 2021-2022 | 2020-2021 | 2019-2020 |
| --- | --- | --- | --- | --- | --- | --- |
| Coinfections (%) | 0 | 0 | 0 | 0 | 0 | 0 |
| Coinfections with RSV (%) | 0 | 0 | 0 | 0 | 0 | 0 |
| Coinfections without RSV (%) | 0 | 0 | 0 | 0 | 0 | 0 |
| 2 viruses  (with RSV) | 0 | 0 | 0 | 0 | 0 | 0 |
| 3 viruses  (with RSV) | 0 | 0 | 0 | 0 | 0 | 0 |
| 4 or more viruses  (with RSV) | 0 | 0 | 0 | 0 | 0 | 0 |

Table 4 severity measures: number of patients with missing data for each aspect considered

|  | 2024-2025 | 2023-2024 | 2022-2023 | 2021-2022 | 2020-2021 | 2019-2020 |
| --- | --- | --- | --- | --- | --- | --- |
| Length of stay (days): Cumulative Per-person median (Q1-Q3) | 0 | 0 | 1 | 2 | 0 | 2 |
| ICU* admission  Number (percentage) | 0 | 0 | 0 | 0 | 0 | 0 |
| ICU length of stay (days):  Cumulative  Per-person median (Q1-Q3) | 0 | 0 | 0 | 2 | 0 | 2 |
| Respiratory support need  Number (percentages) | 1 | 0 | 2 | 0 | 0 | 2 |
| Days on respiratory support: Cumulative  Per-person median (Q1-Q3) | 1 | 0 | 2 | 1 | 0 | 2 |
| Complications  Number (percentages) | 0 | 0 | 1 | 1 | 0 | 2 |

Table 5: Respiratory support use (HFNC, CPAP/NIV, MV) and days on support: number of patients with missing data for each aspect considered

|  | 2024-2025 | 2023-2024 | 2022-2023 | 2021-2022 | 2020-2021 | 2019-2020 |
| --- | --- | --- | --- | --- | --- | --- |
| Low flow oxygen  Number (percentage) | 1 | 2 | 2 | 1 | 0 | 2 |
| Low flow oxygen  Days median (Q1-Q3) | 1 | 2 | 3 | 1 | 0 | 2 |
| HFNC*Number (percentage) | 1 | 2 | 1 | 1 | 0 | 2 |
| HFNC* Days median (Q1-Q3) | 1 | 3 | 1 | 1 | 0 | 2 |
| Room air HFNC  (percentage of HFNC) | 1 | 2 | 1 | 1 | 0 | 2 |
| CPAP/NIV**  (percentage) | 0 | 0 | 0 | 0 | 0 | 1 |
| CPAP/NIV** Days median (Q1-Q3) | 0 | 0 | 0 | 0 | 0 | 1 |
| MV***  (percentage) | 0 | 0 | 0 | 0 | 0 | 0 |
| MV*** Days median (Q1-Q3) | 0 | 0 | 0 | 0 | 0 | 0 |
| Highest FiO2 median (Q1-Q3) | 2 | 3 | 5 | 2 | 0 | 2 |
